# TAP (T and Small Protrusion) and Culotte Stenting Techniques Versus a Reverse Crush in Case of Bailout of Provisional Stenting

**DOI:** 10.3390/jcdd13010024

**Published:** 2026-01-01

**Authors:** Stefano Cangemi, Dario Buccheri, Vittorio Virga, Massimo Benedetto, Federico Giannino, Francesco Stabile, Federico Inglese, Daniele Vinci, Giovanna Geraci

**Affiliations:** 1 Cardiology Unit, S. Antonio Abate Hospital, ASP Trapani, 91016 Erice, Italy; 2 Department of Clinical and Experimental Medicine, Policlinic “G. Martino”, University of Messina, 98100 Messina, Italy; 3 Division of Cardiology, Ospedale Civico Arnas, 90100 Palermo, Italy; 4 Division of Cardiology, Department of ProMISE, University Hospital P. Giaccone, University of Palermo, 90127 Palermo, Italy

**Keywords:** percutaneous coronary intervention (PCI), bifurcation stenting techniques, coronary artery disease (CAD), MACE (major adverse cardiovascular events)

## Abstract

Coronary bifurcation lesions are considered among the most challenging lesions to treat with percutaneous coronary intervention (PCI), particularly in cases of extensively diseased branches. In the event of failure of a provisional (one-stent) approach, many two-stent bifurcation techniques can be performed for the treatment of coronary bifurcation lesions. Two techniques (culotte and T/TAP) are suggested by the European Bifurcation Club, but a reverse crush is performed by some operators. This study aims to retrospectively compare these two different approaches (EBC-recommended techniques vs. reverse crush).

## 1. Introduction

Coronary artery disease (CAD) remains one of the leading causes of death worldwide [1]. Percutaneous coronary intervention (PCI) is the most frequently used method of coronary revascularization, especially in cases of acute coronary syndromes [2]. Coronary bifurcation lesions are considered one of the most difficult lesions to treat with PCI, especially in the case of extensively diseased branches [3]. Numerous recent trials have demonstrated the superiority of the one-stent (provisional) bifurcation technique over an upfront two-stent bifurcation technique, particularly in instances where the side branch is not extensively diseased [4,5]. The European Bifurcation Club (EBC) recommends initially using the “Provisional” technique, followed by a two-stent approach (preferably T/TAP or culotte) in cases where the side branch (SB) may be compromised, as indicated by a reduced TIMI flow, dissection, or severe stenosis [6,7,8]. Some interventional cardiologists prefer to perform a reverse (or internal) crush [9] as a bailout technique of a provisional stent. These techniques have never been compared before.

## 2. Materials and Methods

**Figure 1:**
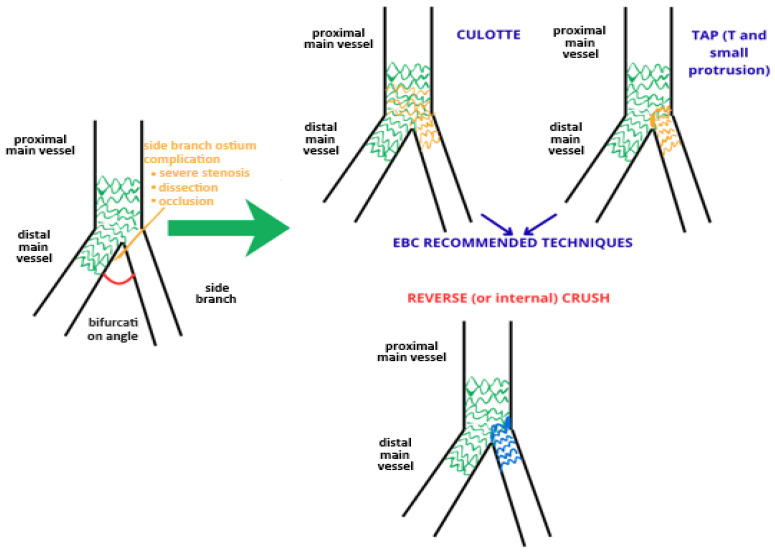
SB stenting in bailout (TAP and culotte vs. reverse crush).

This study aimed to evaluate the benefit of a T/TAP or culotte technique versus a reverse crush in the case of the bailout of a provisional strategy. The primary endpoint was major adverse cardiovascular events (MACEs) during two years of follow-up. We also reported contrast-induced nephropathy (CIN) during hospitalization. This was a retrospective study that evaluated T/TAP or culotte PCI versus reverse crush PCI used as a bailout of a provisional stent performed between 2019 and 2024 at high-volume PCI centers (Figure 1). This study was conducted in catheterization laboratories with expertise in intracoronary imaging, forming part of an acute ST-elevation myocardial infarction (STEMI) H24 network. We enrolled patients affected by coronary artery disease who underwent PCI of a coronary bifurcation lesion with an upfront one-stent strategy, and after a side branch (SB) complication, the operator had to switch to a two-stent strategy. We collected all procedural characteristics: type of bifurcation treated, diameter of branches, balloon and stent used, length of PCI, amount of contrast medium used, and intravascular imaging. Each PCI was accurately analyzed step-by-step and classified according to the MADS-2 classification [6,7], with the aim of understanding whether the PCI followed the EBC recommendation. We evaluated the patients’ baseline characteristics and blood examinations during hospitalization.

The inclusion criteria were as follows:

(1) Hospital admission for acute coronary syndromes or chronic coronary syndromes with an indication for percutaneous coronary revascularization.
(2) A native coronary bifurcation de novo lesion.
(3) A planned coronary bifurcation percutaneous coronary intervention (PCI) utilizing a provisional technique that was subsequently converted to a two-stent bifurcation technique.
(4) A Medina 1.1.1, 1.1.0, or 0.1.1 lesion [10].

**Exclusion criteria**: Platelet count < 100,000 cells/mm^3^; severe hepatic impairment defined as Child–Pugh class C; prior history of hemorrhagic stroke or subarachnoid hemorrhage; history of an intracranial neoplasm, arteriovenous malformation, or aneurysm; or patients who received fibrinolytic therapy within 48 h before the PCI and patients with a history of bleeding diathesis.

**Angiographic exclusion criteria:** Severe tortuosity around the target bifurcation, chronic total occlusions, massive thrombus in left main coronary artery, or a Medina 0.0.1 lesion [10].

**Endpoint definition:**

The primary endpoint of this study was major adverse cardiovascular events (MACEs) [11]. A MACE is an endpoint represented by cardiac death, myocardial infarction, stroke, stent thrombosis, target vessel revascularization, or major bleeding. We also reported the incidence of contrast-induced nephropathy (CIN), even if it was not a primary endpoint. CIN is an iatrogenic renal injury that follows intravascular administration of radio-opaque contrast media (CM) in susceptible individuals [12].

**Description of coronary bifurcation techniques:**

- ***TAP (T and small protrusion) technique [7,8]:***

Here, we describe the TAP technique using the steps described in the last EBC publication [7,8]:

(1) The first step is wiring the side branch and main vessels.
(2) The second step is the implantation of the main vessel stent (or a stent across the distal main vessel if an inverted technique is used); this is sized according to the diameter of the distal main vessel in crossover with the bifurcation.
(3) The third step is the proximal optimization technique (POT), which consists of the dilatation of the proximal tract of the main vessel stent with a balloon sized 1:1 with the proximal main vessel.
(4) Kissing balloon inflation (KBI).
(5) SB stent implantation sized 1:1 with the SB diameter to cover the ostium with minimal protrusion, while in the MV, there is a balloon sized 1:1 with the distal MV uninflated.
(6) A final KBI.
(7) Repeat the POT (optional).

This technique is useful in bifurcations with easy SB access and less than 90 degrees of angulation between the proximal MV and the SB. This technique is useful if the SB diameter is much smaller than the MV diameter.

- ***Culotte technique [7]:***

(1) Wiring of the MV and SB.
(2) Implantation of the main vessel stent.
(3) Proximal optimization technique (POT).
(4) Rewiring of the SB in a position near the carina.
(5) First KBI or SB dilatation.
(6) Implantation of the second stent from the SB to the proximal MV, protruding a few mm.
(7) Second POT using a 1:1 non-compliant (NC) balloon, making sure the POT balloon does not cross the carina point and covers the entire proximal MV segment.
(8) A final KBI.

This technique is useful if the SB diameter is similar to the distal MV diameter and there is an acute (<70°) angle of bifurcation (between the SB and the distal MV).

- ***Reverse (or internal) crush technique [9]:***

(1) A stent is deployed in the main branch, followed by the POT and final kissing inflation toward the side branch.
(2) A second stent is passed into the side branch and a balloon is positioned in the main branch at the level of the bifurcation, sized 1:1 with the distal MV.
(3) Then the side branch stent is retracted two to three millimeters into the main branch and deployed.
(4) After removing the deployed balloon, check the angiography is taken to ensure a good result in the side branch and exclude the need for any additional stent in the side branch.
(5) After confirming this, the side branch wire is removed and the main branch balloon is inflated at a high pressure to crush the proximal edge of the side branch balloon.
(6) Re-crossing of the side branch followed by side branch dilatation and final kissing dilatation is necessary.

This procedure ensures immediate patency of both branches, but this is more laborious than the standard crush technique.

**Statistical Analysis:**

Continuous variables are presented as the mean ± SD. Discrete variables are expressed as counts and percentages. Baseline differences between groups were analyzed for significance using the Mann–Whitney U test for continuous data and the chi-squared test (or Fisher’s exact test where the expected cell value was less than 5) for categorical variables. Multivariable binary logistic regression analyses were performed to identify the correlates of the baseline and procedural characteristics with MACEs. A *p*-value < 0.05 was considered statistically significant. SPSS statistical software, Version 30 (IBM Corp., Armonk, NY, USA) was used for all the statistical calculations.

## 3. Results

**Baseline clinical characteristics:**

We enrolled 42 patients, predominantly male (38 out of 42). The mean age of the participants was 71.3 years. The cohort presented many comorbidities: 30 patients (71%) had type 2 diabetes mellitus, 9 patients (21%) had peripheral artery disease, and 15 patients (35.7%) suffered from severe COPD. The most common admission presentation was NSTEMI, observed in 29 patients (69%), followed by STEMI in 7 patients (16.7%). Additionally, two patients (4.8%) experienced cardiogenic shock. There were no significant differences in age, sex, comorbidities, or presentation type between the two comparison groups: reverse crush and EBC-recommended techniques (Table 1).

**Baseline angiographical characteristics:**

The most treated coronary bifurcation was the left main, with 27 patients (64.28%), followed by the left anterior descending artery–first diagonal in 14 patients (33.33%). There was only one case involving the left circumflex–obtuse marginal artery. The predominant lesion type was Medina 1.1.1, observed in 37 patients (88.1%), while 22 patients had a narrow bifurcation angle of less than 60° (52.4%). A significant proportion of the treated bifurcation lesions were classified as complex according to the Definition Criteria [5] (18 lesions, 42.85%). Additionally, 16 lesions (38.09%) were evaluated as severely calcified based on an angiographic assessment. The side branch lesion length was longer than 10 mm in many cases (29 lesions, 69%). The side branch stenosis was severe (>70% in left main bifurcations and >90% in non-left main ones) in 85.71% (36 lesions). A high SYNTAX score > 32 was present in 13 patients (30.95%), and an intermediate SYNTAX score (23–32) was present in 16 patients (38.095%). Regarding the baseline angiographic characteristics, the two comparison groups differed significantly only for the presence of more patients with a high SYNTAX score (>32) in the reverse crush group (Table 2).

**Procedural characteristics:**

The TAP technique was performed in 15 patients (35.7%) and the culotte technique in 17 (40.5%). Therefore, EBC-suggested techniques were performed in 32 patients (76.2%). The reverse crush was the least performed technique, utilized in only 10 patients, accounting for 23.8% of cases. Mechanical circulatory support, including an intra-aortic balloon pump or microaxial pump, was employed in 6 patients, representing 14.3%. The radial approach was the most common vascular access method, applied in 38 patients, or 90.5%. Advanced calcium modification techniques were used sporadically, with 7 lesions treated via intravascular lithotripsy and one case involving a rotational atherectomy, amounting to 19.04% of the cases treated with these advanced techniques. Intravascular imaging was utilized in 20 cases, representing 47.62%. Notably, intravascular imaging was performed in 50% of the patients who underwent a reverse crush (5 of 10 patients) and in 46.87% of those who received EBC-suggested techniques (15 out of 32 patients). Predilatation of the main branch was performed in nearly all cases (38, 90.50%), while predilatation of the side branch was less common (26, 61.9%). The median time for all PCIs was 70 min (Table 3). The reverse crush group did not differ from the ECB-recommended technique group for vascular access, rate of predilatation, use of intravascular imaging, or advanced calcium modification techniques. The reverse crush was significantly associated with a longer time for rewiring, longer procedures, and more contrast medium use. Both groups had a very high rate of POT and final KBI. We have to note that only 71.87% of the EBC-recommended group procedures (TAP and culotte) followed the EBC recommendations, often avoiding the first kissing balloon inflation or positioning POT balloon across the carina. A very high rate of procedures had a POT balloon position across the carina, which may account for the high incidence of severe side branch (SB) ostial stenosis or SB occlusion following the initial main vessel stent deployment, often necessitating bailout two-stent strategies (71.43%). When comparing the two groups, this issue was significantly more frequent in the reverse crush cohort than in the EBC-recommended techniques group (100% vs. 62.5%, *p* = 0.020).

**Follow-up and outcomes:**

Follow-up data were available for all patients at 24 months. During the follow-up, six patients experienced a target vessel acute myocardial infarction (14.28%), often caused by thrombosis at the ostium of the side branch or at the distal edge of previously implanted stents. In total, at 24 months, 10 patients underwent target vessel revascularization (23.81%). At the two-year follow-up, only one patient had died, which was due to an acute pulmonary edema related to a suspected acute myocardial infarction. During the hospitalization, the occurrence of CIN after PCI was quite high (15 events; 35.71%). During follow-up, there were no episodes of strokes or bleeding requiring hospitalization, one patient underwent hospitalization for pacemaker implantation, two patients were hospitalized for congestive heart failure, and another one was hospitalized for chronic limb treating ischemia. At two years follow-up, EBC-recommended techniques (TAP and culotte) were significantly associated with a lower incidence of target vessel revascularization (9.37% vs. 30%, *p*-value = 0.040) compared with the reverse crush group; in the EBC group, there was also a trend toward a lower incidence of CIN after the PCI, but it was not statistically significant (28.1% vs. 60%; *p*-value = 0.074), and a lower incidence of MACEs was found (18.75% vs. 50%, *p*-value = 0.094) (Table 4).

**TAP technique:**

There was no difference between the two groups for the baseline clinical and angiographical characteristics apart from a significant incidence of higher SYNTAX score > 32 in the reverse crush group (20% vs. 60%, *p*-value = 0.053). The TAP technique compared to the reverse crush was statistically significantly associated with a lower incidence of CIN (6.66% vs. 60%, *p*-value = 0.007), which was probably related to the amount of contrast medium used (214 ± 37 vs. 331 ± 52, *p*-value = 0.05). There was also a lower rate of MACEs and target vessel revascularization in the TAP group (Table 5). Similar to the EBC technique group, the TAP technique group was significantly associated with a quicker time to rewiring (time to rewiring > 10 min; 6.66% vs. 90%; *p*-value = <0.001), significantly fewer POT balloons across the carina (33.33 vs. 100%, *p*-value = 0.006), and a significantly lower use of SB ballooning after MV stent implantation instead of the first KBI (26.66% vs. 60%, *p*-value = 0.013).

**Culotte technique:**

In our study, no significant differences in clinical outcomes were observed between the culotte and reverse crush techniques (Table 6). The baseline clinical characteristics were generally comparable between the two groups, except for a lower prevalence of severely reduced left ventricular ejection fraction (LVEF) in the culotte group (0% vs. 30%, *p* = 0.046). Bifurcation angles were significantly narrower (<60°) in the culotte group compared with the reverse crush group (76.47% vs. 30%, *p* = 0.024), consistent with the preferential use of the culotte technique in lesions with acute bifurcation angles. Moreover, the time required for side branch rewiring was significantly shorter in the culotte group, with fewer cases exceeding 10 min (47.05% vs. 90%, *p* = 0.031).

**Factors associated with MACE rate:**

We identified several factors significantly associated with major adverse cardiovascular events (MACEs), as summarized in Table 7 and Table 8. Among the clinical characteristics, the presence of peripheral artery disease (PAD) was significantly associated with a higher incidence of MACEs (*p* = 0.038), whereas the mode of presentation (NSTEMI) was not (*p* = 0.69). The Definition Criteria for complex bifurcation lesions was strongly correlated with an increased risk of MACEs. Although the use of two-stent techniques was not statistically associated with MACEs, there was a trend toward a higher event rate in the reverse crush group (*p* = 0.064). Conversely, the use of intravascular imaging (IVUS or OCT) to guide the PCI was identified as a strong protective factor (*p* = 0.003). Similarly, performing a final kissing balloon inflation (KBI) after the first stent implantation—rather than isolated side branch dilation—was associated with a lower incidence of MACEs (*p* = 0.048); among the cases treated with EBC-recommended techniques (TAP and culotte), strict adherence to the EBC procedural steps conferred a significant protective effect (*p* = 0.038). We performed a logistic regression of factors significantly associated with MACEs and we found that the only factors independently associated with MACEs were the use of intravascular imaging (protective factor) and the presence of a bifurcation considered complex using the Definition Criteria (risk factor).

## 4. Discussion

Our study was a multicenter retrospective registry involving tertiary high volume catheterization laboratories. We enrolled a small number of patients compared with the fact that the three recruiting centers together perform about 2500 PCIs per year. The extremely low number of urgent not-upfront two-stent stent bifurcation techniques reflect proper procedure planning, with the choice of an upfront two-stent bifurcation technique in most cases of complex bifurcation lesions. The retrospective nature and the small sample size, especially of the reverse crush subgroup, inevitably limited the statistical power of the study and increased the risk of Type II error, so the results of this study should be considered exploratory or hypothesis-generating. In any case, we believe that our study is useful because it describes, in high-volume centers, the incidence and characteristics of not-upfront two-stent bifurcation techniques. Indeed, this is the first descriptive study that compares these techniques in an emergency context and could serve as a basis for a randomized clinical trial or a larger multicenter registry. Patients enrolled were representative of patients treated daily in our catheterization laboratories, with a mean age of about 70 years old and numerous comorbidities. These comorbidities, the older age, and the acute clinical presentation precluded surgical myocardial revascularization. We enrolled very complex bifurcation lesions (left main stem 64.28%; Medina 1.1.1 in 88.1%; 38.09% severely calcified lesions; SB lesion length was longer than 10 mm in 69% of patients) and severe SB stenosis in 85.71% patients. One limitation of this study is the unequal distribution in the two comparison groups (reverse crush and EBC-recommended techniques) of the high-risk SYNTAX score group (>32), which represents the majority in the reverse crush group, so this could cause a bias when comparing the two groups. It should be noted, however, that regarding the Definition Criteria Complex, the distribution is homogeneous in both groups. Another factor distributed non-uniformly between the two comparison groups is “POT balloon across carina.” This factor could cause an alteration of TIMI flow in the side branch, which may eventually have led to the two-stent bifurcation technique. Consequently, this should be taken into consideration in data analysis. This study confirmed that the Definition Criteria Complex [5] accurately identifies patients at high risk of PCI complications and MACEs at follow-up. The incidence of MACEs at the two-year follow-up in the EBC-recommended group (18.75%) was slightly higher than the angiographic-guided PCI groups of recent randomized controlled trials. In our study, we found a very high incidence of MACEs in the reverse crush group at the two-year follow-up, which were driven mainly by target vessel MI and target vessel revascularization. Interestingly, reverse crush had a non-statistically significant trend toward a higher incidence of MACEs. After performing the multivariate analysis, only the use of intracoronary imaging-guided PCI (protective factor) and a complex bifurcation lesion according to the Definition Criteria (risk factor) were independently associated with MACEs. Use of intravascular imaging for guiding the PCI was the strongest protective factor against MACEs. This is not new data; numerous randomized controlled trials demonstrated the superiority of imaging-guided PCI compared with angiographic-guided PCI [13,14] in terms of MACEs. Following the EBC recommendation, it was associated with fewer cardiovascular events in the TAP and culotte techniques. This result is similar to what was demonstrated in the MOBBEM trial [15]. In this preclinical study, it was demonstrated that more frequent use of POT/KBI and adherence to EBC recommendations might reduce the occurrence of post-PCI suboptimal stent configurations [15]. In this study, performing a KBI instead of isolated SB dilatation was significantly associated with reduced MACEs (*p*-value = 0.048) [15,16]. When we compared the EBC-recommended techniques (TAP and culotte) and reverse crush group, we found that the EBC-recommended techniques group was associated with fewer target vessel revascularizations at the two-year follow-up. The EBC-recommended techniques were associated with a faster rate of rewiring, which was especially related to TAP techniques. In fact, the TAP technique does not require rewiring the ostium of the SB covered by two stent struts but requires only one rewiring, significantly shortening the procedure [7,8]. The EBC-recommended techniques were also associated with lower contrast medium use and a shorter procedural time. We hypothesize the following technical explanation for the increased MACE rate in the reverse crush group: in the reverse crush technique, crushed stent struts are often floating inside the proximal main branch lumen; these crushed struts can often incompletely trigger stent thrombosis and restenosis, where only the use of intravascular imaging can detect this imperfection and fix it before ending the PCI [17,18,19]. Numerous studies, both preclinical and clinical, have demonstrated that in the case of two-stent bifurcation techniques, the use of intravascular imaging and following expert technical recommendations allow for obtaining optimal stent configurations and reduced MACEs [13,16]. In our study, the TAP technique was associated with a lower amount of contrast medium use and was associated with a reduced risk of CIN compared with the reverse crush group. In fact, the risk of CIN increases proportionally with the amount of iodine contrast medium administered [20]. Our result regarding the CIN rate must be taken as a hypothesis generator because we did not perform any adjustment of risk stratification for the CIN risk, for example, with the Mehran score [20]. Also, the low use of intracoronary imaging could introduce a bias because a previous study demonstrated a lower incidence of CIN in the IVUS group [21]. This study has numerous limitations: first, it used a registry, not a randomized controlled trial, so there is a selection bias; second, the very small sample size; third, a non-uniform distribution of a few factors (SYNTAX score and POT balloon across carina); fourth, only half of the patients underwent intravascular imaging PCI, which numerous studies have demonstrated is significantly associated with a lower incidence of MACEs and contrast-induced nephropathy [13,14].

Despite these limitations, our study has two important values: first, we enrolled non-highly selected patients but patients with numerous comorbidities representative of common clinical practice; second, we studied a situation (bailout SB stenting in provisional stenting technique) that is relatively common in clinical practice but has still not been studied in a dedicated randomized controlled trial. In fact, this is the first study to compare the two more commonly used techniques in bailout stenting and not-upfront two-stent strategies, like DK-crush.

## 5. Conclusions

This study was the first study to evaluate different two-stent coronary bifurcation techniques in the case of bailout side branch stenting. We found that the reverse crush technique could be inferior to EBC-recommended techniques (TAP and culotte) because it was associated with a statistically significant higher rate of target vessel revascularization, longer procedure times, and larger amounts of contrast medium use based on the univariate analysis. In the multivariate analysis, we found that a bifurcation considered complex based on the Definition Criteria and performing intravascular imaging guided PCI were independently associated with MACEs. The results of this study should be considered at risk of bias given the small sample size, the uneven distribution of some important factors (SYNTAX score, POT balloon across carina) in the two groups under study, and the retrospective and observational nature of the study.

## Figures and Tables

**Table 1:** Baseline clinical characteristics.

	EBC-Recommended Techniques (TAP and Culotte) (n = 32)	Reverse Crush (n = 10)	*p*-Value
Age (years)	70 ± 10	71 ± 8	0.150
Male	30 (93.75%)	8 (80%)	0.236
Hyperlipidemia	27 (84.37%)	9 (90%)	0.759
Hypertension	28 (87.5%)	8 (80%)	0.443
Diabetes	25 (78.12%)	5 (50%)	0.096
Severe COPD	10 (31.25%)	5 (50%)	0.239
Peripheral artery disease	6 (18.75%)	3 (30%)	0.362
STEMI	5 (15.62%)	2 (20%)	0.539
NSTEMI	21 (65.62%)	8 (80%)	0.330
Cardiogenic shock	2 (6.25%)	0 (0%)	0.576
LVEF ≤ 35%	3 (9.37%)	3 (30%)	0.153

COPD, chronic obstructive pulmonary disease; LVEF, left ventricular ejection fraction; NSTEMI, non-ST-elevation myocardial infarction; STEMI, ST-elevation myocardial infarction.

**Table 2:** Baseline angiographic characteristics.

	EBC-Recommended Techniques (TAP and Culotte) (n = 32)	Reverse Crush (n = 10)	*p*-Value
LM bifurcation	21 (65.62%)	6 (60%)	0.513
LAD-D bifurcation	10 (31.25%)	4 (40%)	0.440
LCX-MO bifurcation	1 (3.125%)	0 (0%)	0.762
Severe calcification	12 (37.5%)	4 (40%)	0.585
Angle of bifurcation < 60°	19 (59.37%)	3 (30%)	0.104
SYNTAX score > 32	7 (21.87%)	6 (60%)	0.032
SYNTAX score between 23 and 32	14 (43.75%)	2 (20%)	0.165
Complex Definition Criteria	13 (40.62%)	5 (50%)	0.434
SB lesion length > 10 mm	24 (75%)	5 (50%)	0.136
Severe SB stenosis > 90% no LM and >70% LM	29 (90.62%)	7 (70%)	0.135
Medina 1.1.1	27 (84.37%)	10 (100%)	0.237

D, diagonal; LAD, left anterior descending artery; LCX, left circumflex artery; LM, left main; MO, marginal obtuse; SB, side branch.

**Table 3:** Procedural characteristics.

	EBC-Recommended Techniques (TAP and Culotte) (n = 32)	Reverse Crush (n = 10)	*p*-Value
Radial approach	28 (87.5%)	10 (100%)	0.321
Femoral approach	4 (12.5%)	0 (0%)	0.203
Advanced calcium modification techniques (IVL or rotational atherectomy)	6 (18.75%)	2 (20%)	0.626
Intravascular imaging	15 (46.87%)	5 (50%)	0.574
Predilatation MV	30 (93.75)	9 (90%)	0.679
Predilatation SB	21 (65.62%)	5 (50%)	0.30
Time of rewiring > 10 min	8 (25%)	9 (90%)	0.01
Cause of switch to double-stent technique:			
– Severe stenosis of SB ostium	19 (59.37%)	8 (80%)	0.390
– Dissection of SB ostium	8 (25%)	2 (20%)	0.633
– SB occlusion	5 (15.62%)	0 (0%)	0.415
POT performed	31 (96.87%)	10 (100%)	0.762
First KBI	16 (50%)	4 (40%)	0.249
SB dilatation after first stent	16 (50%)	6 (60%)	0.35
Final KBI	32 (100%)	10 (100%)	0.504
POT balloon across carina	20 (62.5%)	10 (100%)	0.020
EBC recommendations followed	23 (71.87%)	/	/
MV stent/reference distal MV	1	1.016	0.534
SB stent/reference SB	1	0.97	0.518
Pallone POT/reference prox MV	0.95	0.877	0.135
Angiographic success	31 (96.87%)	9 (90%)	0.424
Mechanical circulatory support	3 (9.375%)	3 (30%)	0.135
Procedural time, minutes	65.15 ± 17	89.2 ± 20	0.05
Contrast volume, ml	252 ± 57.7	331 ± 52.4	0.054

IVL, intravascular lithotripsy; KBI, kissing balloon inflation; MV, main vessel; POT, proximal optimization technique; SB, side branch.

**Table 4:** Clinical outcomes at two-year follow-up after using EBC-recommended techniques (TAP and culotte) vs. reverse crush.

	EBC-Recommended Techniques (TAP and Culotte) (n = 32)	Reverse Crush (n = 10)	*p*-Value
Hospitalization			
CIN	9 (28.1%)	6 (60%)	0.074
Dialysis after PCI	2 (6.25%)	0 (0%)	0.576
Two-Year Follow-Up			
MACEs	6 (18.75%)	5 (50%)	0.094
Cardiac death	1 (3.12%)	0	0.205
Target vessel MI	3 (9.37%)	3 (30%)	0.135
Target vessel revascularization (comprehensive for target vessel MI)	5 (15.62%)	5 (50%)	0.040

Values are n (%). The *p*-values are from chi-square tests. CIN, contrast induced nephropathy; MACEs, major adverse cardiovascular events; MI, myocardial infarction; PCI, percutaneous coronary intervention.

**Table 5:** Clinical outcomes at two-year follow-up for TAP technique vs. reverse crush.

	TAP (n = 15)	Reverse Crush (n = 10)	*p*-Value
Hospitalization			
CIN	1 (6.66%)	6 (60%)	0.007
Two-Year Follow-Up			
MACEs	2 (13.3%)	5 (50%)	0.062
Cardiac death	0 (0%)	0 (0%)	0.286
Target vessel MI	1 (6.66%)	3 (30%)	0.159
Target vessel revascularization (comprehensive for target vessel MI)	2 (13.3%)	5 (50%)	0.062

Values are n (%). The *p*-values are from chi-square tests. CIN, contrast induced nephropathy; MACEs, major adverse cardiovascular events; MI, myocardial infarction.

**Table 6:** Clinical outcomes at two-year follow-up for culotte technique vs. reverse crush.

	Culotte (n = 17)	Reverse Crush (n = 10)	*p*-Value
Hospitalization			
CIN	8 (47.05%)	6 (60%)	0.402
Dialysis	2 (11.76%)	0 (0%)	0.387
Two-Year Follow-Up			
MACEs	4 (23.53%)	5 (50%)	0.162
Cardiac death	1 (5.88%)	0 (0%)	0.275
Target vessel MI	2 (11.76%)	3 (30%)	0.249
Target vessel revascularization (comprehensive of target vessel MI)	3 (17.65%)	5 (50%)	0.91

Values are n (%). The *p*-values are from chi-square tests. CIN, contrast induced nephropathy; MACEs, major adverse cardiovascular event; MI, myocardial infarction.

**Table 7:** Clinical, angiographic, and procedural factors significantly associated with MACEs.

	*p*-Value
Peripheral artery disease	0.038
Reverse crush	0.064
Definition Criteria Complex	0.003
EBC technique steps followed (for TAP and culotte)	0.038
Use of intravascular imaging (IVUS or OCT)	0.003
KBI after first stent implantation	0.048

Values are n (%). The *p*-values are from chi-square tests. EBC, European bifurcation club; IVUS, intravascular ultrasound; KBI, kissing balloon inflation; OCT, optical coherence tomography; TAP, T and small protrusion.

**Table 8:** Logistic regression of factors significantly associated with MACEs.

	Coefficient	Standard Error	t-Statistic	Lower 95%	Upper 95%	Probability
Constant	0.1762	0.1447	1.2177	−0.1219	0.4743	0.2347
Use of intravascular imaging (IVUS or OCT)	−0.2067	0.1457	−1.4186	−0.5067	−0.0934	0.0168
Peripheral artery disease	0.1853	0.1641	1.1295	−0.1526	0.5232	0.2694
Definition Criteria Complex	0.3223	0.1342	2.4020	0.0459	0.5986	0.0241
KBI after first stent implantation	−0.1877	0.1636	−1.1472	−0.5247	0.1493	0.2621
EBC technique steps followed (for TAP and culotte)	0.0287	0.1933	0.1485	−0.3694	0.4268	0.8831

## Data Availability

The datasets generated and/or analyzed during the current study are available from the corresponding author upon reasonable request.
